# Deficiency of NEIL3 Enhances the Chemotherapy Resistance of Prostate Cancer

**DOI:** 10.3390/ijms22084098

**Published:** 2021-04-15

**Authors:** Yiwei Wang, Liuyue Xu, Shanshan Shi, Sha Wu, Ruijie Meng, Huifang Chen, Zhenyou Jiang

**Affiliations:** 1 Department of Microbiology and Immunology, College of Basic Medicine and Public Hygiene, Jinan University, Guangzhou 510632, China; ivywong200909@gmail.com (Y.W.); lewisxu95@gmail.com (L.X.); shiss@jnu.edu.cn (S.S.); iccy712@gmail.com (S.W.); mrj201909@gmail.com (R.M.); 2 School of Pharmacy, Guangdong Lingnan Institute of Technology, Guangzhou 510663, China

**Keywords:** NEIL3, prostate cancer, chemotherapy resistance

## Abstract

Acquired treatment resistance is an important cause of death in prostate cancer, and this study aimed to explore the mechanisms of chemotherapy resistance in prostate cancer. We employed castration-resistant prostate cancer (CRPC), neuroendocrine prostate cancer (NEPC), and chemotherapy-resistant prostate cancer datasets to screen for potential target genes. The Cancer Genome Atlas (TCGA) was used to detect the correlation between the target genes and prognosis and clinical characteristics. Nei endonuclease VIII-like 3 (NEIL3) knockdown cell lines were constructed with RNA interference. Prostate cancer cells were treated with enzalutamide for the androgen deprivation therapy (ADT) model, and with docetaxel and cisplatin for the chemotherapy model. Apoptosis and the cell cycle were examined using flow cytometry. RNA sequencing and western blotting were performed in the knockdown Duke University 145 (DU145) cell line to explore the possible mechanisms. The TCGA dataset demonstrated that high NEIL3 was associated with a high T stage and Gleason score, and indicated a possibility of lymph node metastasis, but a good prognosis. The cell therapy models showed that the loss of NEIL3 could promote the chemotherapy resistance (but not ADT resistance) of prostate cancer (PCa). Flow cytometry revealed that the loss of NEIL3 in PCa could inhibit cell apoptosis and cell cycle arrest under cisplatin treatment. RNA sequencing showed that the knockdown of NEIL3 changes the expression of neuroendocrine-related genes. Further western blotting revealed that the loss of NEIL3 could significantly promote the phosphorylation of ATR serine/threonine kinase (ATR) and ATM serine/threonine kinase (ATM) under chemotherapy, thus initiating downstream pathways related to DNA repair. In summary, the loss of NEIL3 promotes chemotherapy resistance in prostate cancer, and NEIL3 may serve as a diagnostic marker for chemotherapy-resistant patients.

## 1. Introduction

Prostate cancer (PCa) is the most commonly diagnosed malignant tumor in the United States, accounting for 21% of all cases of cancer in men [1]. Hormonal therapy is the typical first-line treatment, but PCa can evolve into castration-resistant prostate cancer (CRPC) within 2–3 years [2]. With the advent of new therapeutics, a more aggressive neuroendocrine prostate cancer (NEPC) has become more prevalent [3]. Some PCas spread to other organs, such as the bones, lungs, and even brain, causing metastatic prostate cancer (mPCa) [4]. CRPC, NEPC and mPCa are all considered advanced prostate cancer. Advanced PCa always has a very poor prognosis and high mortality; it is not only resistant to androgen deprivation therapy (ADT), but also insensitive to chemotherapy [5,6,7,8]. Advanced PCa is the main cause of death in PCa patients, and the median survival times for CRPC and NEPC patients are less than 3 years [9,10]. Acquired resistance to treatment is an important cause of death in these patients, so there is an urgent need to identify the mechanism of therapy resistance.

In this study, we employed four reported CRPC, NEPC and chemotherapy-resistant prostate cancer datasets. Common changes in gene expression were screened, and the target gene Nei endonuclease VIII-like 3 (NEIL3) was identified as particularly relevant. NEIL3 is a gene located on human chromosome 4q34.3, which is considered to initiate the first step in base excision repair by cleaving bases damaged by reactive oxygen species and introducing DNA strand breaks via lyase action [11]. However, additional functions of the gene have been identified. It is reported that NEIL3 can promote the repair of interstrand crosslinks [12], drive autoimmunity [13], suppress apuricic/apyrimidinic endonuclease 1 (APE1) [14], etc. Here, we found lower NEIL3 in CRPC and chemotherapy-resistant prostate cancer, and found low NEIL3 to be associated with a poor prognosis. Further study demonstrated that a loss of NEIL3 could cause changes in neuroendocrine-related genes. Moreover, a deficiency of NEIL3 could significantly promote the phosphorylation of ATR serine/threonine kinase (ATR) and ATM serine/threonine kinase (ATM) during chemotherapy, thus initiating downstream pathways related to DNA repair. In summary, this study demonstrated a possible diagnostic marker for clinical chemotherapy-resistant patients.

## 2. Results

### 2.1. NEIL3 Was Associated with Chemotherapy Resistance in PCa

**Figure 1:**
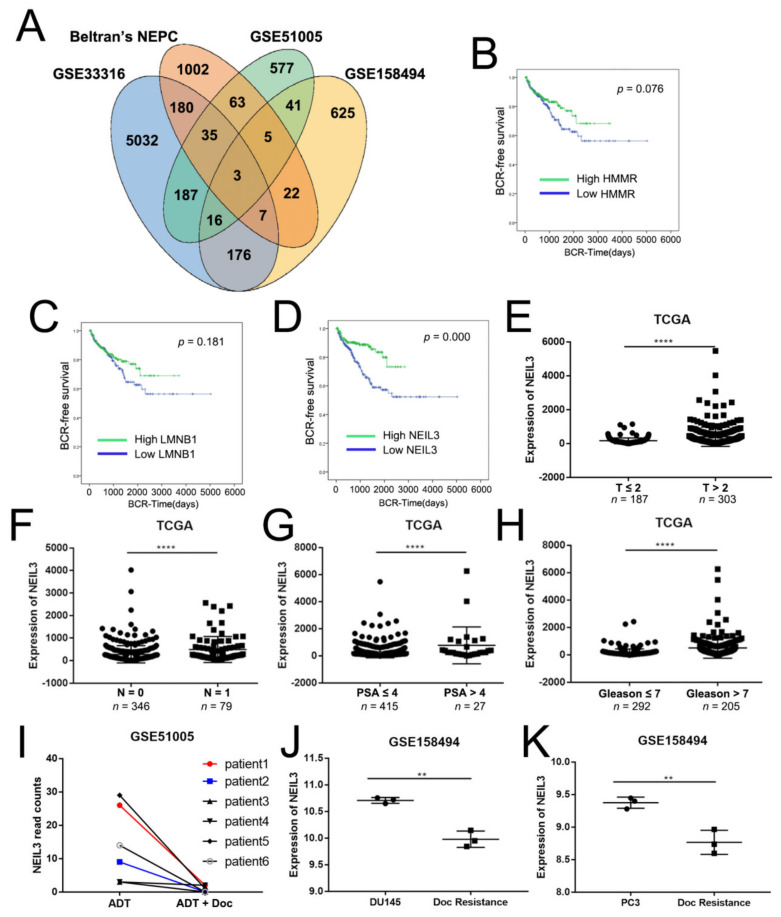
Database-integrated screening identified that NEIL3 showed low expression in therapy-resistant samples. (**A**) Three target genes were selected from the intersection of the castration-resistant prostate cancer (CRPC) dataset, neuroendocrine prostate cancer (NEPC) dataset, and chemotherapy-resistant datasets. (**B**–**D**) Biochemical recurrence (BCR)-free survival analysis for HMMR, LMNB1, and Nei endonuclease VIII-like 3 (NEIL3) in the Cancer Genome Atlas (TCGA) dataset. (**E**–**H**) Correlation of NEIL3 expression with the clinicopathological characteristics of PCa patients in the TCGA dataset. (**I**–**K**) The expression of NEIL3 in 2 chemotherapy-resistant datasets, ** *p* < 0.05 and **** *p* < 0.0001, versus corresponding control group.

Therapy-resistant prostate cancer (PCa) is clinically lethal, and is the leading cause of death from PCa. CRPC and NEPC are two types of PCa that are resistant to current therapies after a period of clinical treatment. We downloaded a CRPC dataset (GSE33316, 5032 genes changed) and Beltran’s NEPC dataset (1002 genes changed) [15] from PubMed, finding 180 genes common to both. To further narrow down the results, we merged the results from two chemotherapy datasets: GSE51005 (577 genes changed) and GSE158494 (625 genes changed). Finally, three candidate genes (Hyaluronan mediated motility receptor (HMMR), Lamin B1 (LMNB1), and NEIL3) related to therapy resistance were selected (Figure 1A). The biochemical recurrence (BCR) survival curves for these three genes demonstrated that only NEIL3 was significantly correlated with the prognosis of PCa (*p* = 0.076, 0.181, and 0.000, respectively; Figure 1B–D). Therefore, we chose NEIL3 as our target gene.

### 2.2. NEIL3 Showed Low Expression in Therapy-Resistant PCa

To further explore the correlation between NEIL3 and the clinicopathological characteristics of PCa, we employed the Cancer Genome Atlas (TCGA) dataset, which included 497 patients. We found that high NEIL3 was associated with a higher T stage (*p* < 0.0001, Figure 1E), N stage (*p* < 0.0001, Figure 1F), prostate-specific antigen (PSA) level (*p* < 0.0001, Figure 1G) and Gleason score (*p* < 0.0001, Figure 1H). The above results suggest that NEIL3 may be an oncogene, but contrarily, NEIL3 was also positively correlated with a good prognosis (Figure 1D). All of the patients from TCGA had received clinical treatment, but the depictions of the BCR-free curves cannot exclude this factor. Therefore, we hypothesized that NEIL3 can promote therapeutic sensitivity. In other words, if the patient is in a natural, untreated state, high NEIL3 may indicate a poor prognosis, but if the patient receives clinical treatment, NEIL3 could promote sensitivity to the therapy, improving the prognosis. To validate this hypothesis, we examined the expression levels of NEIL3 in docetaxel-resistant prostate cancer (GSE51005 and GSE158494). The results indicate that NEIL3 was little expressed in docetaxel-resistant prostate cancer clinical samples (Figure 1I) and cell lines (Figure 1J,K). NEIL3 also showed low expression in CRPC samples (GSE33316) (data not shown). Taken together, we suspected that a loss of NEIL3 may activate therapeutic resistance, thus leading to a poor prognosis in PCa.

### 2.3. Loss of NEIL3 Facilitated Docetaxel and Cisplatin Resistance of PCa, but Could Not Promote ADT Resistance

**Figure 2:**
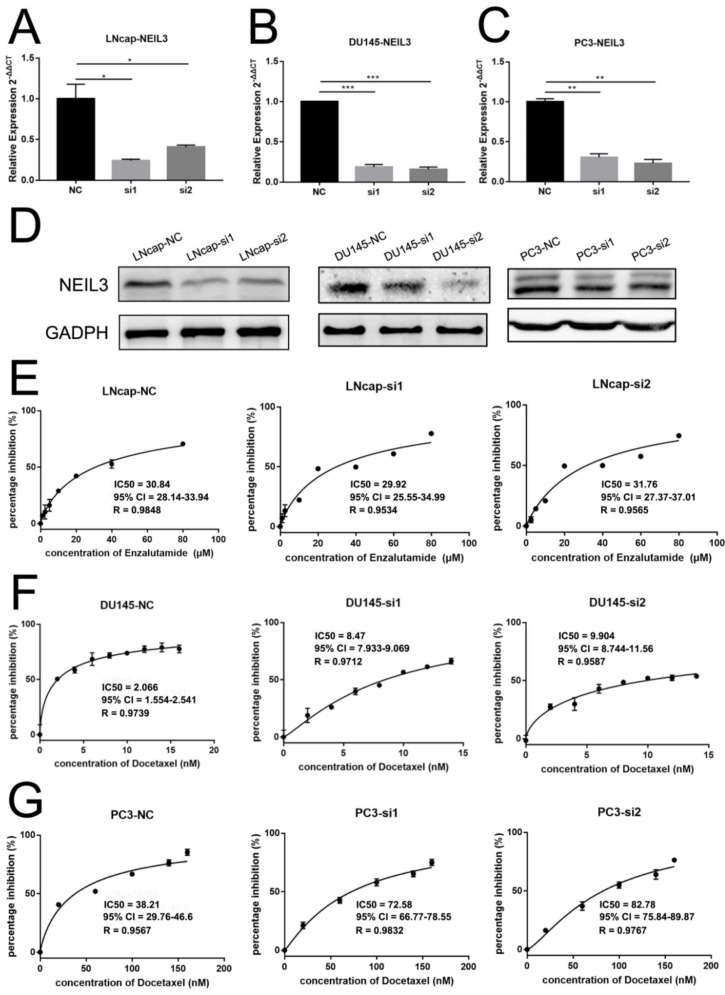
Loss of NEIL3 facilitates docetaxel resistance of DU145 and PC3 but does not affect androgen deprivation therapy (ADT) resistance. (**A**–**D**) qRT-PCR and western blot analysis for NEIL3 expression in NEIL3 knockdown LNcap, DU145 and PC3 cells. (**E**) The MTS assay to determine IC50 of enzalutamide in LNcap cells after treatment. (**F**,**G**) The MTS assay to determine IC50 of docetaxel in DU145 and PC3 cells after treatment, * *p* < 0.05, ** *p* < 0.01 and *** *p* < 0.001, versus corresponding control group.

To verify the role of NEIL3 in therapeutic sensitivity, we constructed NEIL3 knockdown cell lines via transfecting two small interfering RNAs into lymph node carcinoma of the prostate (LNcap), Duke University 145 (DU145) and prostate cancer cell line (PC3) cells. Western blotting and qPCR confirmed that NEIL3 was downregulated at the mRNA and protein levels (Figure 2A–D). We chose enzalutamide, docetaxel, and cisplatin to build an ADT and chemotherapy cell model, because ADT and docetaxel are the first choice for PCa patients. Although cisplatin is not widely used for prostate cancer, it is often applied to NEPC and DNA damage repair mutant patients in the clinic [16,17]. We found that NEIL3 knockdown significantly reduced the sensitivity of PCa cells to chemotherapy. However, NEIL3 did not affect the sensitivity of PCa to ADT.

For the enzalutamide ADT model, in LNcap (Figure 2E), the IC50s of enzalutamide were similar in the three groups: 30.84 μM (NC), 29.92 μM (si1), and 31.76 μM (si2).

For the docetaxel chemotherapy model, in DU145 (Figure 2F), the IC50s of docetaxel were 2.066 nM in the control group, and 8.470 nM (si1) and 9.904 nM (si2) in the NEIL3 knockdown groups. In PC3 (Figure 2G), the IC50s were 38.21 nM in the control group, and 72.58 nM (si1) and 82.78 nM (si2) in the NEIL3 knockdown groups.

**Figure 3:**
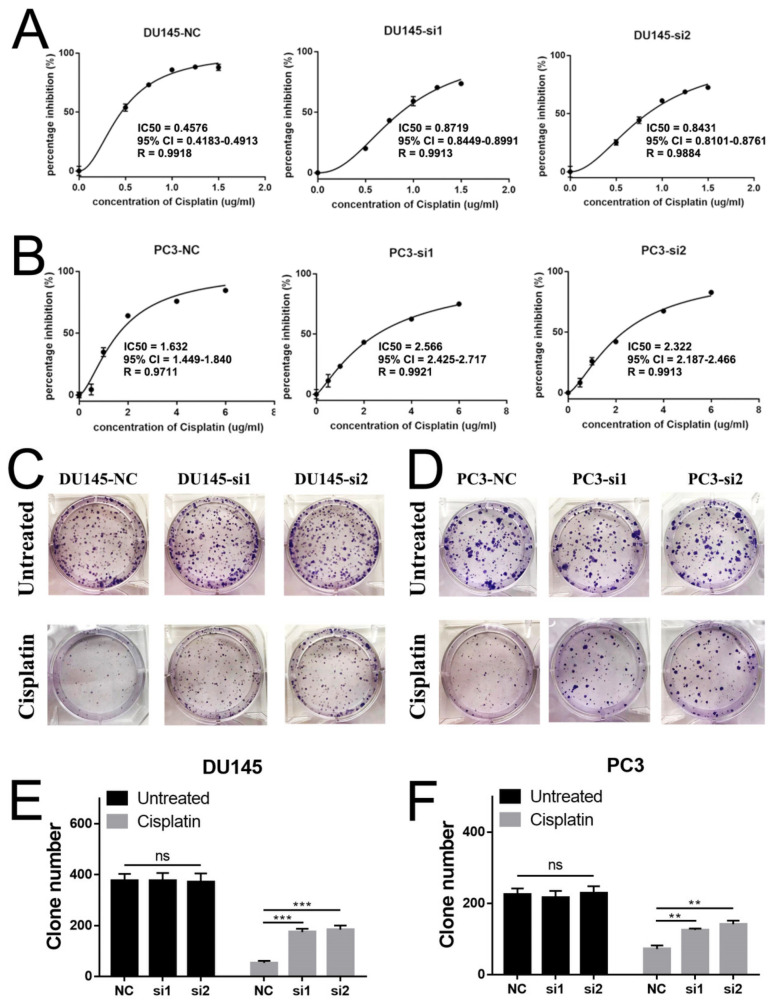
Loss of NEIL3 facilitates cisplatin resistance of DU145 and PC3. (**A**,**B**) The MTS assay to determine IC50 of cisplatin in DU145 and PC3 cells after treatment. (**C**,**D**) Colony formation assay to test cell viability in the DU145 and PC3 cell lines, before and after cisplatin treatment. (**E**,**F**) Statistical analysis of the number of colonies among different groups, ** *p* < 0.01 and *** *p* < 0.001, versus corresponding control group.

For the cisplatin chemotherapy model, in DU145 (Figure 3A), the IC50s of cisplatin were 0.4576 μg/mL in the control group, and 0.8719 μg/mL (si1) and 0.8431 μg/mL (si2) in the NEIL3 knockdown groups. A similar result was found in PC3 (Figure 3B): the IC50s of cisplatin were 1.632 μg/mL in the control group, and 2.566 μg/mL (si1) and 2.322 μg/mL (si2) in the NEIL3 knockdown groups. A colony formation assay was performed to validate these results. We found that the loss of NEIL3 had no effect on the proliferation of PCa cells, but under cisplatin treatment (0.1 μg/mL in DU145 and 0.3 μg/mL in PC3) there were more clones in the NEIL3 knockdown group (Figure 3C–F).

Taking these findings together, it can be seen that the loss of NEIL3 facilitates the docetaxel and cisplatin resistance of PCa cells, but the cause needs to be further explored.

### 2.4. Loss of NEIL3 Facilitates Cisplatin Resistance by Regulating Apoptosis and Cell Cycle Arrest

**Figure 4:**
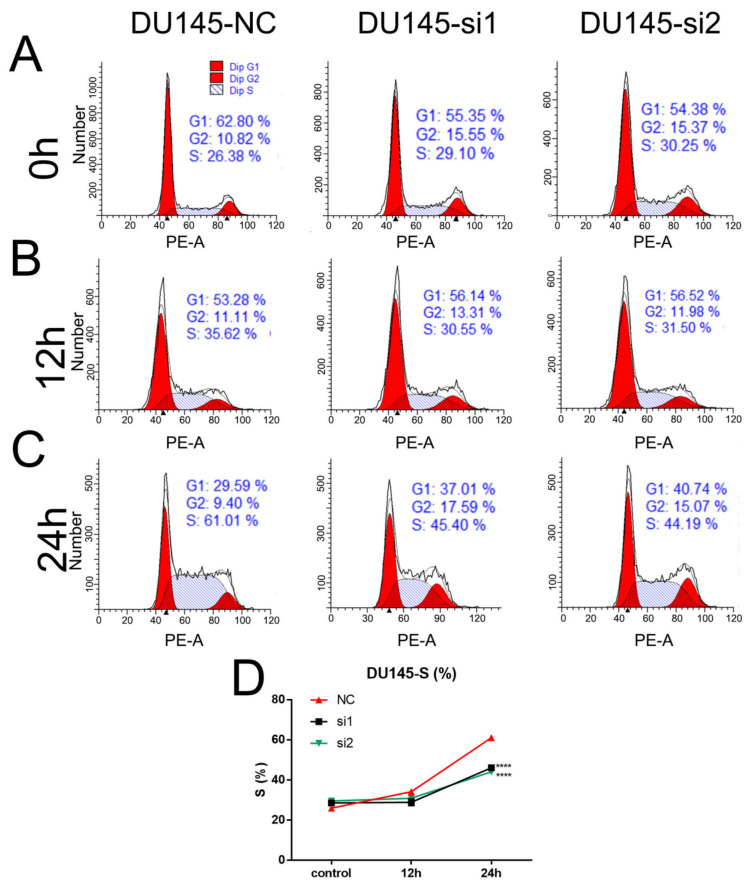
Loss of NEIL3 promotes cisplatin resistance by alleviating S-phase block. (**A**–**C**) Representative flow cytometry cell cycle images of DU145 before and after cisplatin treatment. (**D**) Relative S-phase change curves for DU145 after cisplatin treatment, **** *p* < 0.0001, versus the corresponding time point negative control (NC) group.

**Figure 5:**
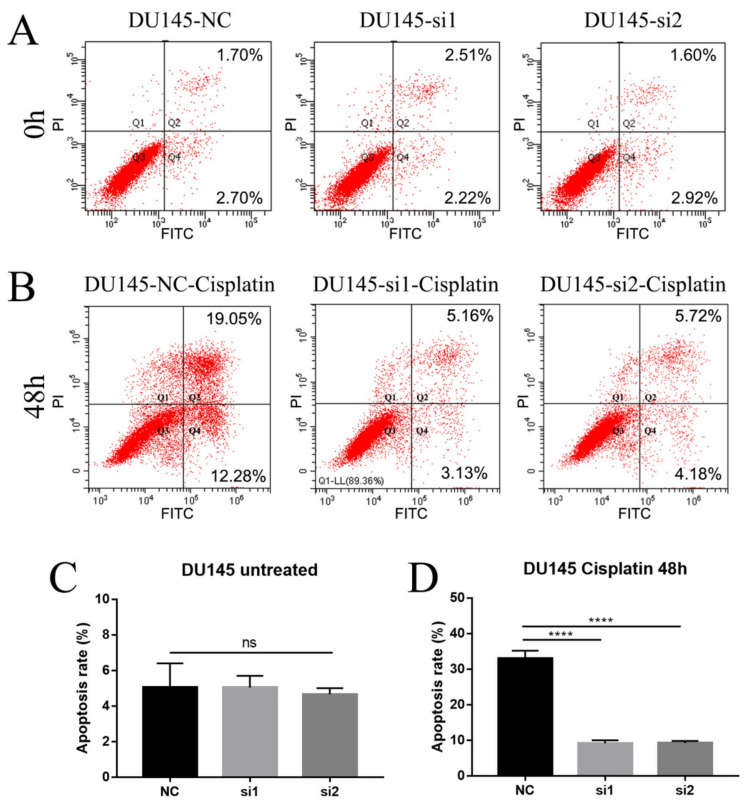
Loss of NEIL3 promotes cisplatin resistance by affecting apoptosis. (**A**,**B**) Representative flow cytometry cell apoptosis images of DU145 before and 48 h after cisplatin treatment. (**C**,**D**) Histogram analysis of apoptotic cell counts of DU145 before and 48 h after cisplatin treatment, **** *p* < 0.0001, versus NC group.

Cell cycle arrest and apoptosis are the two most important factors affecting cell proliferation, and we therefore examined them to explore the possible mechanisms by which NEIL3 could affect chemotherapy sensitivity. Flow cytometry was performed on DU145 cells at 0 h, 12 h and 24 h after cisplatin therapy, and the results indicate that the loss of NEIL3 alone had no effect on cell cycle arrest. DU145 underwent S-phase arrest after chemotherapy (Figure 4A–C), but the extent of the S-phase blockade in the NEIL3 knockdown group was significantly lower than that in the control group (Figure 4D). Under replication stress, the cell cycle is stalled to allow enough time for damaged DNA to be repaired [18]. In other words, cell cycle arrest is a self-protective mechanism, preventing DNA damage from spreading to the next generation. Here, we found that S-phase arrest was reduced by NEIL3 knockdown, suggesting that there was less DNA damage. That means that the loss of NEIL3 may activate the relevant DNA repair mechanism upon cisplatin exposure. To further verify this conjecture, we also measured the level of apoptosis in DU145 after 48 h of cisplatin treatment. We found that the loss of NEIL3 alone had no effect on apoptosis (Figure 5A,C), but PCa cells underwent apoptosis under cisplatin treatment, and the level of apoptosis induced by cisplatin in the NEIL3 knockdown group was significantly lower than that in the control group (Figure 5B,D). Taking these findings together, it can be seen that the loss of NEIL3 may facilitate chemotherapy resistance by activating the relevant DNA repair mechanism.

### 2.5. NEIL3 Affects the Level of Neuroendocrine and Invasion-Related Genes

**Figure 6:**
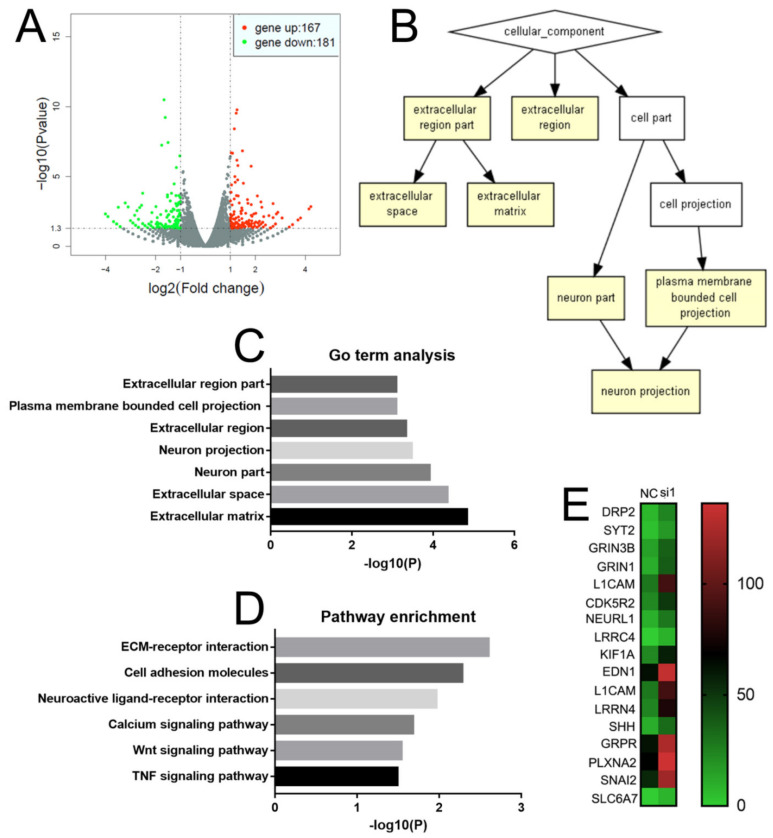
NEIL3 affects neuroendocrine and invasion-related gene changes. (**A**) Volcano map of genes differentially expressed in the NEIL3 knockdown group. (**B**,**C**) Gene ontology (GO) term analysis of differentially expressed genes. (**D**) Kyoto Encyclopedia of Genes and Genomes (KEGG) analysis of differentially expressed genes. (**E**) Heat map comparing neuroendocrine-related genes’ expression between the control and NEIL3 knockdown groups.

In order to explore the role of NEIL3 in PCa, we sent the NEIL3 knockdown and control DU145 cells for RNA sequencing. The results showed that 167 genes were upregulated while 181 genes were downregulated by NEIL3 knockdown (Figure 6A). To further explore the roles of these genes, Gene ontology (GO) term analysis and Kyoto Encyclopedia of Genes and Genomes (KEGG) pathway enrichment analysis were performed. For GO term analysis, we found that the enriched cellular components were important for metastasis and neuronal development (extracellular matrix and neuron projection, Figure 6B,C). A similar result was found by the KEGG pathway analysis (Figure 6D): Extracellular matrix (ECM) receptor interaction, cell adhesion, and molecular and wingless-related integration site (Wnt) signaling pathways are very important for metastasis [19,20,21], while neuroactive ligand–receptor interaction is critical for neuronal development. In addition, some studies have reported that calcium signaling pathways and tumor necrosis factor (TNF) signaling pathways are very important for tumorigenesis and the epithelial–mesenchymal transition (EMT) of PCa [22]. Moreover, we found that many neuroendocrine-related genes were upregulated when NEIL3 was knocked down (Figure 6E). It is well known that the alterations of neuroendocrine- and invasion-related markers are the two most important features of NEPC [23]. Here, we also observed a similar alteration when NEIL3 was knocked down. In other words, a loss of NEIL3 may be involved in the neuroendocrine process of PCa, thus promoting chemotherapy resistance. 

### 2.6. NEIL3 Regulated Chemotherapy Resistance through the ATR Serine/Threonine Kinase (ATR) and ATM Serine/Threonine Kinase (ATM) Pathways

**Figure 7:**
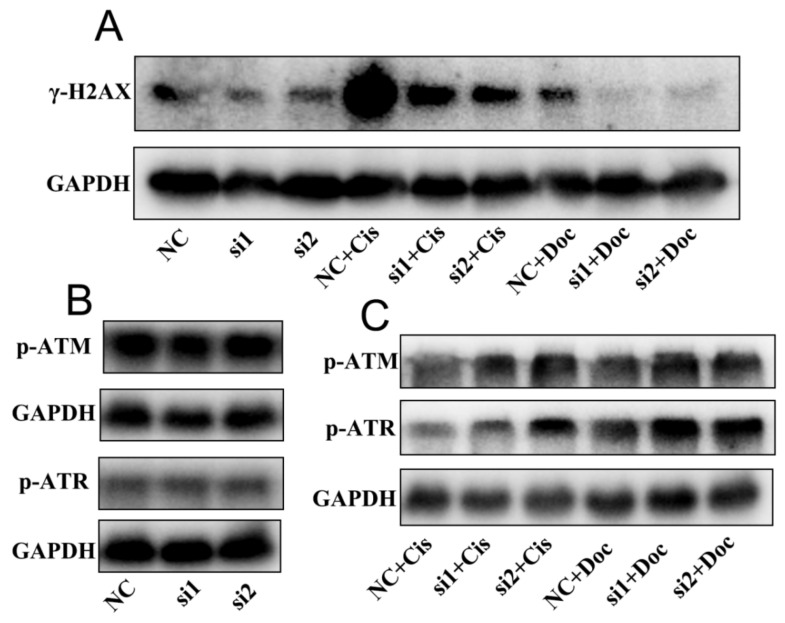
Loss of NEIL3 promotes chemotherapy resistance by affecting p-ATM and p-ATR. (**A**) Western blotting image of γ-H2AX before and after chemotherapy in DU145. (**B**) Western blotting image of phosphorylated ATM and phosphorylated ATR when NEIL3 was knocked down. (**C**) Western blotting image of phosphorylated ATM and phosphorylated ATR after cisplatin and docetaxel treatment. Cis = cisplatin; Doc = docetaxel.

To validate the role of NEIL3 in DNA repair, further western blotting was performed to quantify gamma-H2A histone family, member X (γ-H2AX), a sign of DNA damage. We found that NEIL3 itself had little influence on the level of γ-H2AX, but under cisplatin or docetaxel treatment, the loss of NEIL3 could significantly alleviate DNA damage (Figure 7A). The activation of ATM and ATR is central in the response to DNA damage, and hundreds of downstream proteins are phosphorylated in an ATM- or ATR-dependent manner [24,25]. Therefore, we speculated that NEIL3 might affect DNA repair by regulating the phosphorylation of ATR and ATM. NEIL3 itself did not influence the level of phosphorylated ATR (p-ATR) or phosphorylated ATM (p-ATM), but under cisplatin or docetaxel exposure the loss of NEIL3 significantly promoted the phosphorylation of ATR and ATM (Figure 7B,C), thus initiating downstream pathways related to DNA repair. Taken together, these findings convincingly demonstrate that NEIL3 can regulate chemotherapy resistance through the ATR and ATM pathways.

## 3. Discussion

Acquired resistance to therapy remains the major limitation of therapeutic efficacy in PCa. CRPC and NEPC are the two most typical subtypes of prostate cancer with acquired therapy resistance, and they are also the main factors leading to death in PCa patients. Therefore, based on reported CRPC, NEPC, and chemotherapy-resistant datasets, we screened a target gene, NEIL3, and found its expression to be low in therapy-resistant samples. The TCGA cohort’s data indicated that NEIL3 was correlated with worse clinicopathological characteristics but a good prognosis, which also suggests that NEIL3 may be related to clinical treatment sensitivity. In the present research, we found that the loss of NEIL3 could facilitate chemotherapy resistance. Regarding the mechanism, NEIL3 may be involved in the neuroendocrine process of PCa and the activation of ATR/ATM, thus activating the relevant DNA repair mechanism.

It is interesting that the loss of NEIL3 facilitated resistance to docetaxel and cisplatin but could not promote ADT resistance. ADT has long been used as the standard therapy to treat PCa [26], and it targets androgen receptor (AR) signaling, suppressing the progression of PCa [27]. Here, we found that the loss of NEIL3 promoted chemotherapy resistance mainly through signaling related to DNA repair, but the inhibition of AR could not cause DNA damage directly. The inhibitory effects of docetaxel and cisplatin on PCa have different mechanisms. Although docetaxel was reported to exert its anticancer effect mainly by promoting microtubule assembly and stabilizing microtubule structures [28], some other studies demonstrated that it could also cause a certain degree of DNA damage [29,30]. Cisplatin could interact with purine bases, resulting in the generation of DNA lesions, which finally leads to apoptosis [31]. Therefore, we believe that the loss of NEIL3 confers resistance only to those treatments that cause DNA damage. Another study proved that NEIL3 could facilitate radiotherapy sensitivity [32], which also supports our conjecture. Of note, docetaxel is one of the first-line drugs in the clinic; combining this with a NEIL3 booster is a promising therapy for docetaxel-resistant patients.

NEIL3 belongs to the NEIL family, which is considered to initiate base excision repair to maintain genomic stability [33,34]. Unlike the other members of its family (NEIL1 and NEIL2), NEIL3 has more complex functions, which have not been fully elucidated [12,34,35]. The somatic mutation burden exhibits a significant positive correlation with NEIL3, but a significant inverse correlation with NEIL1 and NEIL2 [36]. Thus, NEIL3 may perform an opposite function compared to NEIL1 and NEIL2. Here, we also found that the loss of NEIL3 may activate the relevant DNA repair mechanism by promoting the phosphorylation of ATR and ATM; these are initially activated in response to DNA damage, and promote DNA repair [25,37]. Many studies have shown that NEIL3 potentially acts upstream of the ATM/ATR pathways. Klattenhoff found that the loss of NEIL3 enhanced sensitivity to ATR inhibitors in glioblastoma cells [38]. Wallace clarified that NEIL3 exhibits high homology with the GRF zinc finger (Zf-GRF) domain, which can activate the ATR pathway [39]. Another study showed that the loss of NEIL3 could directly increase the protein level of ATR, thus accelerating the phosphorylation of ATR [32]. In summary, we determined that NEIL3 regulated chemotherapy resistance by influencing the phosphorylation of ATR/ATM.

NEPC is the most malignant prostate cancer clinically, with an average survival time of less than 1 year [40]. Importantly, NEPC can tolerate almost all existing treatments [41]. Clinical therapies mainly rely on cisplatin-based chemotherapy [42], but NEPC quickly develops cisplatin resistance and causes tumor-specific death [43,44]. Here, we found that NEPC showed lower NEIL3, and knocking down NEIL3 could increase the levels of neuroendocrine-related markers. Thus, NEPC may exhibit faster DNA repair than other types of PCa, and these mechanisms need to be explored in more depth.

A limitation of this study is that we only constructed a knockdown system, because no NEPC cell model suitable for constructing an overexpression model has been described to date. The existing NEPC cell lines generally grow in suspension (such as neuroendocrine PCa cell line NCI-H660), making it difficult to perform the overexpression procedure on them, and cell function experiments are also difficult to carry out. Our future experiments will focus on building an appropriate NEPC cell model to validate the role of NEIL3. Moreover, although the results suggested that NEIL3 did not affect the sensitivity of PCa to ADT, we detected a low level of NEIL3 in CRPC. It is necessary to show the silencing and enhancing of NEIL3 in a CRPC model and determine if it could alter androgen receptor (AR) function.

## 4. Materials and Methods

### 4.1. Database Analysis

We downloaded 3 datasets from the Gene Expression Omnibus to screen for possible target genes (https://www.ncbi.nlm.nih.gov/geo/query/acc.cgi, accessed on 1 November 2020): the CRPC dataset (GSE33316), clinical chemotherapy dataset (GSE51005), and chemotherapy resistance cell line dataset (GSE158494). In addition, the NEPC dataset from Beltran’s research was also used [15]. Genes commonly showing differential expression across the four databases were taken as the potential target genes. Statistical product and service solutions 20.0 (SPSS 20.0) was used to depict the biochemical recurrence (BCR)-free survival curves for the target genes according to the TCGA database, for further screening. We analyzed the correlation between NEIL3 and patients’ clinicopathological features based on the TCGA database (497 patients in total).

### 4.2. Transient Transfection to Knock Down NEIL3 in DU145 and PC3

Small interfering RNA (siRNA) oligonucleotides were purchased from GenePharma (Shanghai, China). The siRNA sequences were as follows: si1 sense (5′–3′), GCAGGACUUGCUCUCUCUATT; si1 antisense (5′–3′), UAGAGAGAGCAAGUCCUGCTT; si2 sense (5′–3′), GCAAGCUACCGACUAGAAATT; si2 antisense (5′–3′), UUUCUAGUCGGUAGCUUGCTT. Briefly, one day before transfection, 3 × 10^5^ cells were plated in 2 mL of growth medium in a 6-well plate such that they would reach 60–80% confluence by the next day. To transfect each well, 5 µL of RNAimax was diluted in 125 µL of opti-MEM, and 3 µL of 20 µM siRNA was diluted in 125 µL of opti-MEM. The diluted RNAimax (Invitrogen Corporation, Carlsbad, CA, USA) was combined with the diluted siRNA, and they were mixed by vortexing and then incubated for 5 min at room temperature. RNAimax siRNA complexes were added to each well containing cells, which were harvested 48 h after transfection for further use.

### 4.3. RNA Isolation and qPCR

Total RNA was isolated according to standard procedures. Briefly, the cells were suspended and counted, and then pelleted by centrifugation. The supernatant was removed, and the cells were resuspended in 1 mL of RNAiso Plus (TaKaRa Bio, Shiga, Japan). The cells were lysed by pipetting them up and down, and the homogenized samples were incubated for 5 min at room temperature. Then, 0.2 mL of chloroform was added per 1 mL of reagents; the mixtures were shaken vigorously by hand for 15 s and then incubated for 2–3 min. They were then centrifuged at 12,000 bpm for 15 min at 4 °C, and three layers were expected to appear. The top layer (0.5 mL) was transferred to a clean tube and supplemented with 1 μL of RNase-free glycogen and 0.5 mL of RNase-free isopropanol. The samples were centrifuged at 12,000 bpm for 10 min at 4 °C in order to pellet the RNA. The supernatant was removed, the RNA pellet was washed twice with 1 mL of 75% ethanol, and the tubes were centrifuged again at 12,000 bpm for 5 min. The RNA pellet was air dried and then resuspended in RNase-free water, followed by RNA analysis using the NanoDrop 2000 (Thermo Scientific, Waltham, Massachusetts, USA).

The PrimeScript Reverse Transcription Master Mix kit (Takara, Japan) was used to synthesize the complementary DNA. Quantitative real-time PCR was carried out using the TB Green Premix Ex TaqII kit (TaKaRa Bio, Japan) in a CFX96 real-time PCR system. The forward primer for NEIL3 was GCAGTAAACACAACCGCCTC, and the reverse primer was AAGGACAAATCTGCCCATTCAA.

### 4.4. Western Blotting

The proteins from the cultured cells were harvested by using a phenylmethylsulfonyl fluoride (PMSF) lysate buffer. Samples containing 50 μg of protein were separated by 10% sodium dodecyl sulfate-polyacrylamide gel electrophoresis (SDS–PAGE) and then transferred to polyvinylidene fluoride membranes. After the membranes were blocked with 5% non-fat milk for 1 h, they were incubated with the primary antibody at 4 °C overnight: NEIL3 (ab230908; Antibody Cambridge (Abcam)), p-ATR (2853S; Cell Signaling Technology (CST)), p-ATM (5883S; CST), γ-H2AX (9718S; CST), or glyceraldehyde-3-phosphate dehydrogenase (GAPDH) (2118S; CST). The membranes were then incubated with the horseradish peroxidase (HRP)-labeled secondary antibody (Proteintech, Wuhan, Hubei, China) at 37 °C for 1 h. The protein band signal was detected using the BeyoECLPlus chemiluminescence reagent (Beyotime, Shanghai, China).

### 4.5. Cytotoxicity Assay and Colony Formation Assay

The MTS assay (Promega, Beijing, China) was used to determine the IC50s of enzalutamide, docetaxel, and cisplatin (Selleck, Shanghai, China) in LNcap, PC3, and DU145. Briefly, cells were seeded in 96-well plates with gradated concentrations of the therapeutic drugs, and then the 96-well plates were incubated at 37 °C for 48 h. The absorbance at 492 nm was measured by using a microplate reader (TECAN Spark 10 M, Shanghai, China). A total of 1000 cells/well were seeded in 6-well plates, and clones were harvested after 14 days of culture. Then, the colonies were fixed with 4% paraformaldehyde and then stained with 0.2% crystal violet. The number of clones was counted for statistical analysis.

### 4.6. RNA Sequencing and Data Analysis

RNA samples were sent to RiboBio Co., Ltd. Guangzhou, China, for sequencing. Kyoto Encyclopedia of Genes and Genomes (KEGG) biological pathway (https://www.genome.jp/, accessed on 1 December 2020) and GO term analysis (http://cbl-gorilla.cs.technion.ac.il/, accessed on 1 December 2020) were performed for the differentially expressed genes.

### 4.7. Flow Cytometry

Apoptosis and the cell cycle were assayed via flow cytometry (Beckman CytoFLEX, Brea, CA, USA). A total of 4 × 10^5^ cells were washed twice with PBS before being resuspended in 100 μL of 1× binding buffer and 5 μL of Annexin V-FITC (Transgen biotech, Guangzhou, China). Then, the samples were incubated with 5 μL of propidium iodide (PI) for 15 min before apoptosis was measured. For the cell cycle, 4 × 10^5^ cells were washed twice with phosphate buffer saline (PBS) and then fixed with chilled 75% ethanol overnight. Then, the cells were incubated with 50 μL of RNase and 300 μL of PI for 30 min before flow cytometry. The CytExpert 2.0 (Beckman, Brea, CA, USA) and ModFit LT5.0 (Verity Software House, Topsham, ME, USA) were used to analyze the apoptosis and the cell cycle, respectively.

### 4.8. Statistical Analysis

All the quantitative data are presented as the mean ± SD, and were assessed by one-way analysis of variance followed by Student’s t test (GraphPad, La Jolla, CA, USA). SPSS 22.0 was used to depict the BCR-free survival in the TCGA dataset; *p* < 0.05 was considered statistically significant.

## 5. Conclusions

Prostate cancer tumors develop protective mechanisms against the current treatments in their “evolution”. Here, we found that the loss of NEIL3 activates chemotherapy resistance, potentially via activation of the relevant DNA repair mechanism. NEIL3 may serve as a diagnostic or therapeutic target for chemotherapy-resistant patients.

## Data Availability

The datasets generated during the current study are available from the corresponding author on reasonable request.

## Abbreviations

ADT: androgen deprivation therapy; AR: androgen receptor; BCR-free survival: biochemical recurrence-free survival; CRPC: castration-resistant prostate cancer; NEIL3: Nei endonuclease VIII-like 3; NEPC: neuroendocrine prostate cancer; PCa: prostate cancer; PSA: prostate-specific antigen; RT-PCR: quantitative reverse transcriptase polymerase chain reaction; siRNA: RNA interference; TCGA: The Cancer Genome Atlas; WB: Western blot.
